# Ligneous amendments increase soil organic carbon content in fine-textured boreal soils and modulate N_2_O emissions

**DOI:** 10.1371/journal.pone.0284092

**Published:** 2023-08-10

**Authors:** Kenneth Peltokangas, Subin Kalu, Karoliina Huusko, Jimi Havisalmi, Jussi Heinonsalo, Kristiina Karhu, Liisa Kulmala, Jari Liski, Mari Pihlatie

**Affiliations:** 1 Department of Agricultural Sciences, University of Helsinki, Helsinki, Finland; 2 Finnish Meteorological Institute, Helsinki, Finland; 3 Institute for Atmospheric and Earth System Research (INAR), University of Helsinki, Helsinki, Finland; 4 Department of Forest Sciences, University of Helsinki, Helsinki, Finland; 5 Department of Microbiology, University of Helsinki, Helsinki, Finland; 6 Department of Agricultural Sciences, Viikki Plant Science Centre (ViPS), University of Helsinki, Helsinki, Finland; 7 Helsinki Institute of Life Science (HiLIFE), University of Helsinki, Helsinki, Finland; Universiti Teknologi Petronas: Universiti Teknologi PETRONAS, MALAYSIA

## Abstract

Organic soil amendments are used to improve soil quality and mitigate climate change. However, their effects on soil structure, nutrient and water retention as well as greenhouse gas (GHG) emissions are still poorly understood. The purpose of this study was to determine the residual effects of a single field application of four ligneous soil amendments on soil structure and GHG emissions. We conducted a laboratory incubation experiment using soil samples collected from an ongoing soil-amendment field experiment at Qvidja Farm in south-west Finland, two years after a single application of four ligneous biomasses. Specifically, two biochars (willow and spruce) produced via slow pyrolysis, and two mixed pulp sludges from paper industry side-streams were applied at a rate of 9–22 Mg ha^-1^ mixed in the top 0.1 m soil layer. An unamended fertilized soil was used as a control. The laboratory incubation lasted for 33 days, during which the samples were kept at room temperature (21°C) and at 20%, 40%, 70% or 100% water holding capacity. Carbon dioxide (CO_2_), nitrous oxide (N_2_O) and methane (CH_4_) fluxes were measured periodically after 1, 5, 12, 20 and 33 days of incubation. The application of ligneous soil amendments increased the pH of the sampled soils by 0.4–0.8 units, whereas the effects on soil organic carbon content and soil structure varied between treatments. The GHG exchange was dominated by CO_2_ emissions, which were mainly unaffected by the soil amendment treatments. The contribution of soil CH_4_ exchange was negligible (nearly no emissions) compared to soil CO_2_ and N_2_O emissions. The soil N_2_O emissions exhibited a positive exponential relationship with soil moisture. Overall, the soil amendments reduced N_2_O emissions on average by 13%, 64%, 28%, and 37%, at the four soil moisture levels, respectively. Furthermore, the variation in N_2_O emissions between the amendments correlated positively with their liming effect. More specifically, the potential for the pulp sludge treatments to modulate N_2_O emissions was evident only in response to high water contents. This tendency to modulate N_2_O emissions was attributed to their capacity to increase soil pH and influence soil processes by persisting in the soil long after their application.

## 1 Introduction

Intensive agricultural practices have promoted the loss of native soil organic carbon (SOC) to an extent where the productivity of the surface soil has declined [1,2], while also threatening the abundance of soil microbiota [3] and contributing to climate change [4]. However, agricultural management practices that enhance soil carbon (C) sequestration can improve soil fertility and mitigate climate change [5–7]. One way of transforming agricultural soils from a C source to a sink is by amending them with non-native C inputs, such as agricultural residues, recycled organic waste, or biochar. These have been shown to increase soil C content along with water and nutrient availability [8,9]. Despite their potential benefits, organic soil amendments may also induce undesirable effects such as increasing carbon dioxide (CO_2_) emissions, which can be produced as part of the decomposition of the organic amendments themselves [10] or as a consequence of increased decomposition of native SOC, a mechanism also known as positive priming [11–13]. Another potential source of greenhouse gas (GHG) emissions is denitrification [14,15], which can increase nitrous oxide (N_2_O) emissions in response to nutrients and wet soil conditions [16,17]. The complexity of interactions arises from the fact that organic soil amendments can provide both C and nutrients while also influencing soil conditions like soil moisture and even temperature [18].

At present, biochar is considered the most promising organic soil amendment for long-term C sequestration [19–21]. This is due to its recalcitrant nature, as pyrolysed C is known to persist in soil over the long-term [22–24]. In addition, the large specific surface area and highly porous structure of biochar have been shown to benefit soil fertility by improving nutrient and water retention, as well as soil structure [25,26]. However, the long-term residual effects of biochar are still unknown, partly due to the lack of appropriate field experiments [27]. The current literature has shown that application of biochar has the potential to reduce soil N_2_O emissions [15,28–32]. However, other studies have found transient or no discernible effects [28,33,34], or even occasional increases in N_2_O emissions [35–38]. According to a several meta-analyses, the application rate, chemical composition, soil pH and soil texture are all important factors in determining soil response to biochar [15,31]. However, until the specific mechanisms underlying the observed effects are known, significant uncertainties remain in assessing the full impact of biochar use in local climate and soil properties [17,19,39].

Finland has one of the greatest forest covers of European countries, which is intensively utilized for its wood resources. This is common in many northern regions where the forest industry is a prominent part of the national economy and energy production. This means that pyrolysis of wood into biochar could be implemented as a means for the Finnish government to achieve its goal to reach C neutrality by 2035 [40]. Conversion of wood-based biomass to highly stable biochar and its application to soil has been getting attention in Finland as one of the potential mechanisms to achieve this ambitious goal [41,42]. Evergreen conifers–*Picea abies* and *Pinus sylvestris*–are the dominant tree species in Finland and could be a great source of feedstock for biochar. On the other hand, the production volume of willow (*Salix spp*.*)* biomass is smaller compared to evergreen conifers, but because of its rapid growth its potential as an energy crop has been explored extensively [43]. Furthermore, when cultivated on marginal land [43,44], willow biochar could be used to simultaneously sequester C and to increase soil quality, making it an attractive feedstock option [45]. Even though both spruce and willow biomass are composed of lignocellulose, both have their distinct physico-chemical characteristics that may influence biochar properties associated with water and nutrient availability [46–48]. Going forward with biochar production and utilization in Finland, it is important to determine the advantages of available feedstock materials to accurately assess their value for farmers and producers alike.

In addition to wood, large quantities of other ligneous biomasses are produced annually. For example, in Finland, the paper and pulp industry produces almost as much biomass as wastewater treatment [49]. The produced effluent sludge is divided into three categories: primary sludge, secondary sludge, and de-inking sludge. The average ratio of primary sludge to secondary sludge is 70:30, but this may vary between mills [50]. Both primary and de-inking sludge are composed of short lignocellulose fibres and mineral fillers (e.g. kaolin or calcium carbonate) whereas secondary sludge is primarily composed of microbial biomass from the wastewater treatment process [51]. The lignin-rich biomass of primary and de-inking sludge can persist in soil for the long-term [24] whereby it can improve soil physico-chemical properties, similar to pyrolysed biomass [52–54]. Yet, the potential for pulp sludge to be used as a soil amendment remains to be ascertained [55], and for the time being, such biomasses are still primarily burnt or landfilled [22,56].

The purpose of this study was to assess the residual effects of a single field application of ligneous soil amendments for soil structure, water retention, and GHG exchange two years after their application to the soil. We hypothesized that ligneous soil amendments would increase SOC content by introducing recalcitrant C into the soil, therefore improving soil porosity by affecting the packing of soil particles, and also significantly altering soil pH due to the liming effect. As a consequence, we hypothesized that ligneous soil amendments would alter the water retention and gas flow in soil, which are decisive factors for soil GHG exchange. To test our hypotheses, we conducted a laboratory incubation experiment with soil samples collected from a soil-amendment field experiment, where ligneous soil amendments were added to the soil two years before the incubation started. In the laboratory, we periodically measured the GHG production rates of incubated soil samples adjusted to four different moisture contents: 20%, 40%, 70%, and 100% soil water holding capacity (WHC). To our knowledge, this is the first time that biochar and pulp sludge and their effects on soil porosity and GHG exchange have been compared. Furthermore, the current study is among the few that have been conducted using boreal fine textured agricultural soil, which make up most of the cultivated soils in Finland [57], therefore improving our understanding of the effects of soil amendment use in northern agriculture.

## 2 Materials and methods

### 2.1 Study site and soil amendments

Soil for this laboratory experiment was collected from a soil-amendment field experiment established in autumn 2016 at Qvidja Farm in south-west Finland (60° 17′ 44″ N 22° 23′ 35″ E). According to the World Reference Base (WRB) the soil texture was clay, consisting of 54% clay, 34% silt and 12% sand [58], and the soil was classified as Vertic Endogleyic Stagnic Cambisol (clayic) [59]. Before the application of soil amendments, the soil was determined to have an average SOC content of 2.4%, carbon to nitrogen ratio (C:N-ratio) of 8.8, and soil pH of 6.4.

The field had been tilled with a mouldboard to around 0.2 m until 2012, and was under no-till or conservation tillage practices until the start of the experiment in 2016. The amendment treatments were established in a randomized block design with three replicate blocks, each treatment taking up one 9 m x 20 m plot in each block. A single application of the ligneous soil amendments was conducted by manually spreading the biomasses on the soil surface, and then harrowing them into the top 0.1 m soil layer in autumn 2016. After application of the soil amendments, tilling was limited to approximately 0.1 m, in order to limit the thickness of the affected soil layer and prevent dilution. In May 2018, the field was first tilled down to approximately 0.05 m, and then sown with oat (*Avena sativa L*. cultivar Matty) using a seeder (Överum Tive CD1830). More information on yields can be found in Kalu et al. [32]. Before 2018, the field had been cultivated for wheat (5 years), caraway (3 years), sugar beet with oilseed rape (2 years), and grass (5 years). During the experiment, all plots also received N-P-K fertilizer (compound fertilizer Yara Mila 3, 23-3-8, Yara Suomi Oy, Helsinki, Finland) at a rate of 80 kg N ha^-1^ per year.

The soil amendments included in this study were: 1) pulp fibre sludge from pulp and paper mill wastewater (FibreS); 2) a lime-stabilized mixed pulp mill sludge, a commercial product by Soilfood Oy, Helsinki, Finland (LimeS); 3) willow (*Salix* spp.) biochar (WilB) produced via slow pyrolysis at 450°C; and 4) spruce (*Picea abie*s) biochar (SprB), produced similarly at 450°C. The amendment treatments were compared to an unamended control (C80N). More precise description regarding the production of the studied ligneous pulp sludge is provided in Rasa et al. [54]. Each of the soil amendments were produced from ligneous, i.e., wood-derived feedstock and were applied to soil as a semi-dry mass. However, because of the distinct differences in composition that made comparison based on dry weight difficult, we focused on application rates varying between ca. 9–22 Mg ha^-1^ in dry weight (Table 1). Therefore, the application rates of FibreS, LimeS, WilB and SprB corresponded to approximately 1.2%, 0.8%, 1.9%, and 1.7% of soil weight, respectively, calculated to the incorporation depth (0.1 m) and assuming soil bulk density (BD) of 1.18 g cm^-3^. Similar rates have been frequently utilized in soil amendment experiments and are generally recommended in literature [15,25,28,56].

**Table 1:** The abbreviations and application rates of the soil amendments incorporated to soil in Qvidja during 2016, as well as the total added carbon, nitrogen, dissolved nitrogen, calcium (Ca), magnesium (Mg), potassium (K), and sodium (Na) contents. Other material properties of the applied amendments include acidity (pH), electrical conductivity (EC), bulk density (BD), and Brunauer-Emmett-Teller surface area (BET) for biochar treatments alone.

	Fibre sludge (FibreS)	Lime-stabilized pulp sludge (LimeS)	Willow biochar (WilB)	Spruce biochar (SprB)
**Application rate** **(Mg ha**^**-1**^ **dw)**	14	9	22	20
**Total C input** **(Mg ha** ^**-1**^ **)**	5.5	3.3	18	19
**Total N input** **(Mg ha** ^**-1**^ **)**	0.006	0.09	0.37	0.09
**C:N-ratio (m/m)**	950	37	44	203
**Water soluble N input (kg ha** ^**-1**^ **)**	0.14	0.27	0.31	0.10
**Ca input (kg ha** ^**-1**^ **)**	696	689	377	217
**Mg input (kg ha** ^**-1**^ **)**	18	8.0	48	18
**K input (kg ha** ^**-1**^ **)**	3.5	2.8	211	64
**Na input (kg ha** ^**-1**^ **)**	13	7.9	14	1.2
**pH (1:5 H**_**2**_ **O)**	9.2	8.9	9.8	8.3
**EC (mS m** ^**-1**^ **)**	5.4	17	30	9.4
**BD (g cm** ^**-3**^ **)**	0.39	0.40	0.23	0.22
**BET (m**^**2**^ **g**^**-1**^)*	–	–	1.3	328

*measured by N_2_ adsorption using Micromeritics 3Flex (Norcross, Georgia, USA).

### 2.2 Soil sampling and storage

The soil-amendment field was sampled in October 2018, following the harvest in August. The bulk soil was collected from the three replicate plots using an open-face Edelman clay auger to a depth of 0.1 m, and combined to form one composite sample for each treatment, which were then stored at 4°C. Within the next week, the bulk soil was sieved (Ø 5 mm) and carefully spread to air-dry at laboratory conditions (22°C). The residual water content in the air-dried soil samples was 5% (m/m), determined by further drying three subsamples overnight at 105°C. The incubation experiment and all chemical analyses were conducted from the composite bulk soil samples as analytical replicates either before or after drying.

Soil BD and related physical properties were determined from 18 undisturbed soil cores (d = 73 mm, h = 48 mm, V = 0.20 L) per treatment, taken in October 2018. Of those 18 soil cores, six were taken per plot: three from the surface (0–0.05 m) and three below the managed soil layer (0.20–0.25 m). The deeper soil layer was sampled to estimate spatial heterogeneity of the experimental field. Sampling was done with an approximate line pattern from the centre of the plots from a single dug pit. The cylinders were then sealed and stored at 4°C until further analyses in January 2019.

### 2.3 Soil properties and carbon content

Soil dry matter content was determined gravimetrically by drying soil samples overnight at 105˚C. Soil pH and electrical conductivity (EC) were measured from the composite bulk soil sample with three analytical replicates (N = 3) using a pH meter Consort C860 (Topac Inc., Cohasset, MA, US) from a 1:2.5 (v/v) soil-water suspension [60]. Soil mineral-N (NH_4_^+^/NO_3_^–^) was determined using 2.0 M KCl (1:5 v/v) extraction (1 h 200 rpm) with six analytical replicates (N = 6) of the fresh bulk soil. Extracts were filtered through 150 mm grade 3-HW folded filters (Sartorius, Gottingen, Germany) and stored frozen (-20°C) before measuring with an automated ion analyser Lachat QuikChem 8000 (Zellweger Analytics, Milwaukee, WI, USA).

Total soil C and N were determined from air-dried composite bulk soil, which was first crushed with a pestle and mortar and then analysed from four analytical replicates (N = 4) using a varioMAX CN analyser (Elementar Company, Langenselbold, Germany). Because the soil was assumed to contain negligible amounts of carbonates, total soil C was assumed to equal SOC content (i.e., *C*_*tot*_ = SOC). The C-stock (*C*_*stock*_, Mg m^-2^) was calculated for a 0.1 m-thick soil layer as follows:

$$C_{stock}=C\;\rho_{bd}(1-g)z_{h}$$ (1)

where *C* is SOC content (g kg^-1^), *ρ*_*bd*_ is dry BD (kg m^-3^), *g* is proportion of gravel (>2 mm), and *z*_*h*_ is thickness of the soil layer (0.1 m). In the further analysis, we express the C-stock in Mg ha^-1^.

The recovery rate (*R*), i.e., the proportion of undecomposed amendment C at the time of sampling, was estimated from the difference between the C-stock measured for treatment *i* and the unamended control divided by the initial C input:

$$R=\frac{\left(C_{stock\;i}-C_{stock\;control}\right)}{C_{input\;i}}\times 100\%$$ (2)

Soil particle density (*ρ*_*s*_) was determined using a pedotransfer function similar to Schjønning et al. [61], but developed for Finnish soils by Heinonen [62], and calculated as follows:

$$\rho_{s}=2.7+0.0007x_{1}-0.0416x_{2}$$ (3)

where *x*_*1*_ is soil clay content (%) and *x*_*2*_ is SOC content (%).

Soil water holding capacity (WHC) was determined gravimetrically by filling a funnel (fitted with filter paper) with soil and saturating it with water, then draining it overnight and weighing three subsamples (N = 3) of 5 g before and after drying them overnight at 105°C. Soil moisture contents (*θ* m/m) at matric potentials (*ψ*) 0.0, -0.3, -6.0, -250 and -1500 kPa were derived using soil water retention curves. The first three pressure points of the water retention curve were determined using the kaolin sandbox method (Eijkelkamp Agrisearch equipment, the Netherlands), and the final two points using pressure plate extractors (Soilmoisture Equipment Corp., Santa Barbara, CA, USA) connected to a compressor (Kaeser Kompressoren, Coburg, Germany) via pressure manifold (Soilmoisture Equipment Corp.). Afterwards, soil samples were dried at 105°C to determine soil BD. Total soil porosity (*φ*) was calculated according to Eq 3 and using the previously determined *ρ*_*bd*_ and *ρ*_*s*_.


$$\varphi =1-\rho_{bd}/\rho_{s}$$ (4)

The soil water retention data was fitted using the van Genuchten (van Genuchten 1980) function in its bimodal form [63] using *Solver* in Microsoft Excel V 16. The fitted data was used to determine the pore size distribution divided into four pore size classes: macropores >30 μm; micropores; 5.0–30 μm; ultramicropores 0.02–5.0 μm; and cryptopores <0.02 μm using the capillary rise equation of Young-Laplace [64], where *d* is the pore neck diameter in μm, and *ψ* is the matric potential in kPa (Eq 4). The largest pore size class was taken to represent structural pores, which are strongly related to BD and compaction [65–67], whereas the smaller pore size classes represent different textural pores and provide habitats for soil microorganisms [68]. Finally, cryptopores are mainly associated with water and gas adsorption.


d=300/Ψ (5)

### 2.4 Soil microbial biomass

We determined microbial biomass carbon (MBC) and microbial biomass nitrogen (MBN) using chloroform fumigation extraction following Vance et al. [69] with modifications of Blagodatskaya et al. [70]. The analysis was conducted from fresh homogenized (*θ* = 29% m/m) bulk soil using four analytical replicates before the incubation experiment as well as for the incubated soil samples after the experiment (N = 4). Briefly, one fumigated and one unfumigated sample from each incubation bottle were extracted with a solution of 0.05 M K_2_SO_4_ (30 min 200 rpm). The extracts were filtered through Whatman No. 42 filter paper and frozen (-20°C). Thawed extracts were filtered with 0.45 μm Minisart syringe filters (Sartorius, Gottingen, Germany) to remove any precipitates before analysing the extracts for dissolved organic C and N using a TOC-V analyser (TOC V Total Organic Carbon analyser, Schimadzu, Kyoto, Japan). The MBC and MBN were calculated by subtracting the concentrations of dissolved organic C and total dissolved N in the control samples from that of chloroform fumigated samples, respectively. Only three analytical replicates were used for statistical analyses to keep an equal number of samples after removing negative values as outliers.

### 2.5 Incubation set-up

The incubation experiment was established in November 2018. Four analytical replicates (N = 4) of 50 g of the previously sieved and air-dried homogenised soil were rewetted to 20%, 40%, 70% and 100% WHC (m/m). The wetted soil samples were transferred to 500 mL glass bottles. The total weight of the bottle was recorded for regular adjustment of soil moisture (S1 Appendix in S1 File), which was carried out after each gas sampling to avoid potential immediate effects that the watering might have on microbial activity [71,72].

The soil samples were incubated in the laboratory at a temperature of 21±0.4°C and relative air humidity of 24±7.0% for 33 days (S2 Appendix in S1 File). During the incubation, bottles were kept uncapped, except during the gas sampling, and stored covered by a frame of black plastic bags to keep them in darkness.

### 2.6 Measuring and calculating soil GHG emissions

The GHG flux measurements were conducted 1, 5, 12, 20 and 33 days after initial adjustment of the soil moisture. Before sampling, each bottle was flushed for 10 sec with ambient air from the laboratory’s air-main, and then closed with an airtight rubber cap and closer. The bottles were then over-pressurized with 50 mL of ambient air. Four gas samples, 20 mL each, were taken from each incubation bottle with a syringe and needle and injected into a 12 mL helium-flushed evacuated gas vial (Labco Limited, Ceredigion, UK). The first sample was taken 1 hour after closing, and the following samples at 5, 9 and 24 hours.

Gas samples were measured for their CO_2_, CH_4_ and N_2_O concentrations using a gas chromatograph (7890A, Agilent Technologies, California, USA) equipped with a flame ionisation detector (FID) and a methaniser for CO_2_ and CH_4_, and an electron capture detector (ECD) for N_2_O [73]. The gas fluxes (*F* μg g^-1^ soil h^-1^) were determined using linear regression fit of gas concentration versus time (*t*) in hours elapsed after enclosing the bottle, using Eq 6 as follows:

$$F=\frac{dC}{dt}\;V\;M_{c}\;\frac{1}{V_{m}}\frac{1}{m_{s}}\frac{T_{0}}{\left(T_{0}+T\right)}$$ (6)

where

*dC* is the concentrations (ppm) of the respective gas (i.e. CO_2_, N_2_O or CH_4_)

*dt* is the elapsed time (h) during sampling

*V* is the volume (m^3^) of the incubation bottle

*M_c_* is the molar mass (g mol^-1^) of the respective gas (i.e. CO_2_, N_2_O or CH_4_)

*m_s_* is the mass (g) of air-dried soil within the incubation bottle

*V_m_* is the volume of one mole of gas at standard temperature and pressure (0.0224 m^3^ mol^-1^)

*T*_0_ is 273 K and

*T* is the incubation temperature (°C).

For easier comparison, all fluxes were transformed to CO_2_ equivalents (CO_2-eq_) according to their global warming potential: 25 in the case of CH_4_, and 298 in the case of N_2_O [74]. The five flux measurements were then used to interpolate the CO_2_, N_2_O, and CH_4_ emissions during the interval periods by assuming that the fluxes changed linearly during the period between two consequent measuring times, e.g., between days 1 to 5, between days 5 to 12, etc. Finally, the GHG emissions calculated for the four intervals were summed up to estimate *CO*_*2-tot*_, *N*_*2*_*O*_*tot*_, and *CH*_*4-tot*_ (*S* mg CO_2-eq_ g^-1^ soil) emissions over the entire 33-day-long incubation period using Eq 7, which were then analysed individually and together (*GHG*_*tot*_).

$$S=\sum_{i=1}^{4}\frac{\left(F_{i}+F_{i+1}\right)}{2}(t_{i+1}-t_{i})$$ (7)

where

*F_i_* is the gas flux (μg CO_2-eq_ g^-1^ soil h^-1^) measured during time *i* and

*t*_*i*+1_−*t_i_* is the time interval between the measurements in hours.

### 2.7 Statistical analysis

All statistical analyses were carried out in IBM SPSS Statistics 28. We used *p*<0.05 as a criterion for statistical significance. All data were tested for homogeneity of variances (Levene’s test) and normality (Shapiro-Wilk test). The mean soil properties were analysed with one-way ANOVA, using Tukey post hoc test for multiple comparisons.

When analysing MBC and MBN, singular negative values were excluded from further analyses due to failed extraction or because the values had been below the detection limit. To preserve an equal number of samples, a random replicate was removed from the other sets, reducing the number of samples from the original four to three (i.e., N = 3). If more than one sample had to be removed (i.e., N<3), the treatment was excluded completely from further statistical analyses. This occurred primarily in the driest soil moisture (20% WHC).

In the GHG data, a total of two replicates out of 80 were removed as outliers when the data point was located 1.5 times the interquartile range above the upper quartile or below the lower quartile. Furthermore, the N_2_O data was subjected to logarithmic transformation in order to meet the normality requirements during data analysis. Pearson correlation tests were carried out to identify the relationship between GHG and soil physical, hydrological and chemical properties.

## 3 Results

### 3.1 Soil chemical properties

All amended soils exhibited higher (*p*<0.05) soil pH than the unamended control (C80N) (Table 2). Moreover, the pH values of the soil samples collected in October 2018 correlated positively (R^2^ = 0.99, *p*<0.001) with the pH values of the ligneous amendments applied to the experimental field in 2016. Furthermore, the increase in soil pH compared to C80N correlated (R^2^ = 0.95, *p* = 0.045) with the sum of cation inputs (Ca, Mg, K, and Na) associated with the amendments applied in 2016. The EC values of the treatments did not differ significantly from C80N (*p*>0.05).

**Table 2:** The mean ± SE soil pH, electrical conductivity (EC), mineral nitrogen (NO3– and NH4+), total nitrogen (N_tot_), and total carbon (C_tot_) contents determined on a dry weight basis from bulk soil samples (depth 0–0.1 m) collected in October 2018, and C-stock determined to a soil depth of 0.1 m. The recovery rate of soil amendments two years after application is also indicated. Statistical differences are indicated by lowercase letters. For abbreviations, see Table 1.

	C80N	FibreS	LimeS	WilB	SprB
**pH (1:2.5 H**_**2**_ **O)**	6.02^a^ ±0.16	6.77^b,c^ ±0.01	6.67^b,c^ ±0.01	6.87^c^ ±0.01	6.44^b^ ±0.00
**EC (mS cm** ^**-1**^ **)**	49.5^a^ ±5.57	62.9^a^ ±1.04	66.5^a^ ±3.93	58.8^a^ ±1.82	48.3^a^ ±4.53
**NO**_**3**_^–^ **(mg kg**^**-1**^**)**	1.89^a^ ±0.02	2.38^b^ ±0.06	2.65^b^ ±0.10	3.26^c^ ±0.11	13.0^d^ ±0.69
**NH**_**4**_^**+**^ **(mg kg**^**-1**^**)**	0.84^a^ ±0.15	0.63^a^ ±0.08	0.87^a^ ±0.06	0.53^a^ ±0.04	0.73^a^ ±0.10
**N**_**tot**_ **(%)**	0.32^a^ ±0.01	0.36^b^ ±0.01	0.35^a,b^ ±0.01	0.35^a,b^ ±0.01	0.37^b^ ±0.00
**C**_**tot**_ **(%)**	2.49^a^ ±0.02	2.57^a,b^ ±0.02	2.62^b^ ±0.01	2.97^c^ ±0.02	3.54^d^ ±0.03
**Soil C-stock (Mg ha** ^**-1**^ **)**	29.1^a^ ±0.27	29.9^a,b^ ±0.25	30.6^b^ ±0.15	37.4^c^ ±0.31	40.7^d^ ±0.37
**Recovery**	–	15.8^a^ ±0.05	45.7^a^ ±0.04	46.4^a^ ±0.02	61.2^a^ ±0.02

All of the amended soils had greater NO_3_^–^ concentrations than C80N (*p*<0.05). Moreover, both biochar treatments exhibited elevated NO_3_^–^ concentrations compared to the other treatments, and SprB had almost seven times the NO_3_^–^ concentration of C80N. Therefore, NO_3_^–^ data was subjected to logarithmic transformation in order to meet the normality requirements during data analysis. No differences (*p*>0.05) were observed for NH_4_^+^ concentrations (Table 2).

All of the amended soils had greater SOC contents (i.e., C_tot_) than C80N, except for FibreS (Table 2). The SOC content of the pooled control soil (C80N) corresponded to a soil C-stock of 29.1 Mg ha^-1^ when calculated for the amended surface soil (0–0.1 m depth). Soils treated with LimeS, WilB or SprB had significantly greater (*p*<0.05) C-stocks compared to the control soil (Table 2). The average recovery rates of these amendments (i.e., the proportion of un-decomposed amendment C remaining in the soil at the time of sampling) corresponded to approximately 45.7%, 46.4%, and 61.2%, respectively. The FibreS treatment did not increase soil C concentration, and the recovery rate of FibreS was only 15.8% (Table 2), indicating very fast initial decomposition after application. Both, the SOC content and the soil C-stocks correlated positively (R^2^ = 0.89, *p* = 0.04 and R^2^ = 0.97, *p* = 0.01) with the original C inputs (Table 1).

### 3.2 Soil porosity

No significant differences (*p* = 0.054) were observed in soil WHC (m/m) determined to a soil depth of 0.1 m. However, all of the amended soils (FibreS, LimeS, WilB, SprB) had slightly elevated average WHC (±SE) at 65.4% ±0.3, 62.7% ±1.8, 62.2% ±0.6, and 66.3% ±1.2, respectively, whereas C80N had WHC of 61.0% ±1.6.

All soils had an equal total porosity (*φ)* in the amended soil layer (0–0.05 m), except for WilB, which had significantly smaller *φ* (*p* = 0.034) when using the experimental blocks as a covariate (Table 3). There were no statistical differences in the pore size distribution between different treatments (Table 3), but the data indicates a trend whereby WilB, and SprB increased the number of ultramicropores (Ø 0.02–5.0 μm). Data on the unamended soil layer (0.20–0.25 m) can be found in S3 Appendix in S1 File.

**Table 3:** Total porosity (φ) and pore size distribution divided into proportion of macropores (>30 μm), micropores (5.0–30 μm), ultramicropores (0.02–5.0 μm) and cryptopores (<0.02 μm). Statistical differences between treatments are indicated by lowercase letters. For abbreviations, see Table 1.

	C80N	FibreS	LimeS	WilB	SprB
***φ* (%, v/v)**	54.7^a^ ±0.98	54.8^a^ ±0.83	54.7^a^ ±1.25	50.8^b^ ±1.03	54.7^a^ ±0.77
**Macropores >30 μm (%)**	19.3^a^ ±3.31	16.0^a^ ±2.37	19.1^a^ ±2.57	13.9^a^ ±2.70	15.0^a^ ±2.21
**Micropores 5.0–30 μm (%)**	3.85^a^ ±1.20	4.51^a^ ±1.21	2.41^a^ ±1.62	4.67^a^ ±0.76	5.64^a^ ±1.90
**Ultramicropores 0.02–5.0 μm (%)**	43.9^a^ ±2.12	45.8^a^ ±1.12	42.2^a^ ±4.11	49.0^a^ ±2.08	49.9^a^ ±2.17
**Cryptopores <0.02 μm (%)**	33.0^a^ ±1.52	33.7^a^ ±1.18	34.0^a^ ±3.01	32.5^a^ ±1.51	29.4^a^ ±1.16

**Fig 1:**
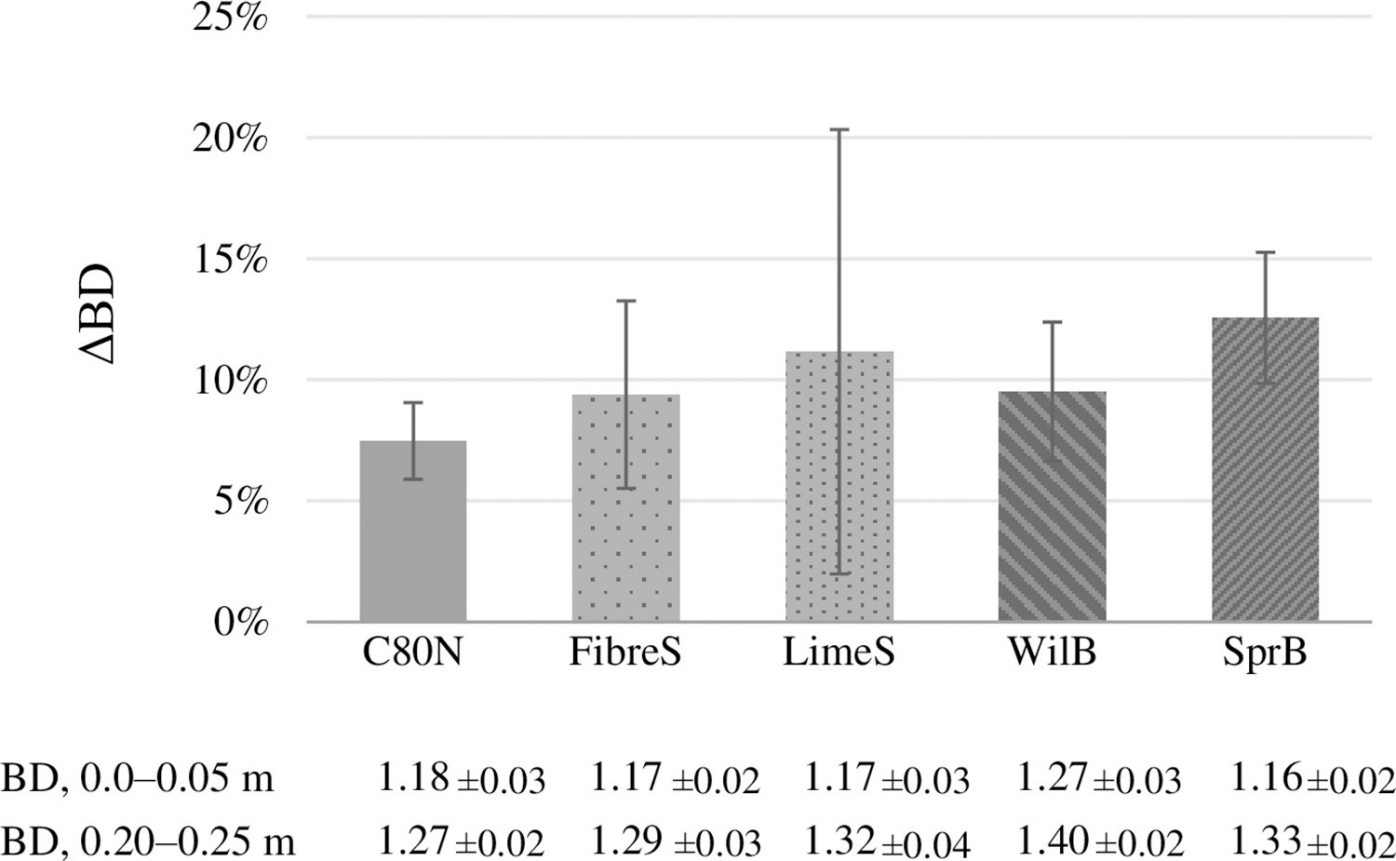
Change in soil bulk density (ΔBD) between the amended soil layer (0–0.05 m) and the deeper unamended soil layer (0.20–0.25 m).

When comparing the BD of the surface soil alone, no significant differences between treatments were shown. However, all treatments exhibited significantly lower BD (*p*<0.05) at the amendment soil layer (0–0.05 m) than at the unamended subsoil (0.20–0.25 m). The difference (ΔBD) in the case of FibreS, LimeS, WilB, and SprB, was 9.4%, 11.2%, 9.5%, and 12.6% (Fig 1), respectively, whereas tillage (control) only reduced it by 7.5%. However, the ΔBD of the amendment treatments were not significantly (*p*>0.05) different from C80N except for WilB, which exhibited significantly higher BD (*p*<0.05) than the other treatments. In addition, the change in ΔBD between the two soil layers correlated positively with the recovery rates of the ligneous soil amendments (R^2^ = 0.94, *p* = 0.02) and were associated with the increased SOC (S4 Appendix in S1 File).

### 3.3 Soil microbial biomass carbon and nitrogen

The statistical differences in MBC, MBN and microbial C:N-ratio between treatments were limited to soil samples taken from the undried bulk soil (Table 4). All amended soils had greater MBC (*p*<0.05), while FibreS, LimeS and WilB also had significantly greater MBN (*p*<0.05) compared to C80N. In addition, both MBC and MBN of the fresh bulk soil had a strong positive correlation with soil pH (R^2^ = 0.92, *p* = 0.02 and R^2^ = 0.81, *p* = 0.10), but the correlation was statistically significant only with MBC. Furthermore, the soil MBC:C_tot_−ratios calculated from samples taken before the incubation were 8.2, 11.1, 11.7, 10.0, and 7.7 whereas the MBN:N_tot_−ratios were 8.4, 10.2, 10.9, 9.7, and 8.3 for C80N, FibreS, LimeS, WilB, and SprB, respectively.

**Table 4:** The mean ± SE microbial biomass carbon (MBC), microbial biomass nitrogen (MBN) and microbial C:N-ratio determined from field-moist bulk soil before incubation started and at the end of the incubation of the rewetted soils (moisture content 20%, 40%, 70% and 100% WHC). Statistical differences are indicated by lowercase letters. For abbreviations, see Table 1.

	Moisture	C80N	FibreS	LimeS	WilB	SprB
**MBC** **(mg kg** ^**-1**^ **)**	Start	204^a^ ±14.9	286^b^ ±11.7	308^b^ ±10.2	296^b^ ±6.37	272^b^ ±5.24
	20%	305^a^ ±36.9	204^a^ ±73.3	366*	288^a^ ±89.7	103*
	40%	146^a^ ±16.8	246^a^ ±82.5	210^a^ ±76.7	193^a^ ±87.2	145^a^ ±67.9
	70%	180^a^ ±50.6	164^a^ ±13.4	217^a^ ±54.6	112^a^ ±39.2	277^a^ ±185
	100%	444^a^ ±120	309^a^ ±80.7	123^b^ ±102	250^a^ ±61.3	304^a^ ±63.1
**MBN** **(mg kg** ^**-1**^ **)**	Start	27.2^a^ ±1.23	36.8^c^ ±1.06	38.7^c^ ±1.69	33.6^b,c^ ±1.25	30.8^a,b^ ±0.53
	20%	1.46^a^ ±0.13	2.34^a,b^ ±0.50	4.34*	7.17^b^ ±0.89	1.94*
	40%	5.95^a^ ±2.64	15.3^a^ ±3.89	11.5^a^ ±5.33	8.99^a^ ±1.05	9.60^a^ ±7.56
	70%	13.9^a^ ±3.68	13.0^a^ ±3.00	8.97^a^ ±3.64	14.7^a^ ±2.70	20.2^a^ ±4.30
	100%	22.9^a^ ±2.15	28.9^a^ ±3.00	28.8^a^ ±1.46	29.0^a^ ±3.68	23.2^a^ ±5.96
**Microbial** **C:N-ratio**	Start	7.47^a^ ±0.22	7.78^a^ ±0.10	8.01^a^ ±0.62	8.85^a^ ±0.53	8.83^a^ ±0.31
	20%	209^a^ ±10.9	116^a^ ±69.2	98.4*	38.9^a^ ±10.0	48.2*
	40%	33.0^a^ ±10.2	23.6^a^ ±14.8	25.5^a^ ±11.9	24.8^a^ ±14.1	19.4^a^ ±11.5
	70%	15.1^a^ ±6.03	13.4^a^ ±2.00	35.0^a^ ±20.3	8.80^a^ ±3.8	18.1^a^ ±12.5
	100%	18.7^a^ ±3.74	10.4^a^ ±2.30	4.49^b^ ±3.81	8.36^a^ ±1.08	11.9^a^ ±5.37

*excluded from statistical analyses due to low number of applicable replicates (N<3).

The differences in microbial biomass contents between moisture treatments were generally limited to the moisture extremes, i.e., 20% and 100% WHC, which exhibited larger MBC compared to 40% and 70% WHC (S5 Appendix in S1 File), whereas MBN increased gradually as the water content of the soil samples increased. In addition, MBN had the second highest correlation with CO_2_ fluxes (R^2^ = 0.78, *p*<0.05) across all moisture levels, after soil moisture (R^2^ = 0.96, *p*<0.05) (S6 Appendix in S1 File). In contrast, MBC did not exhibit any relation with the determined soil moisture properties or soil GHG emissions.

### 3.4 Soil greenhouse gas emissions

**Fig 2:**
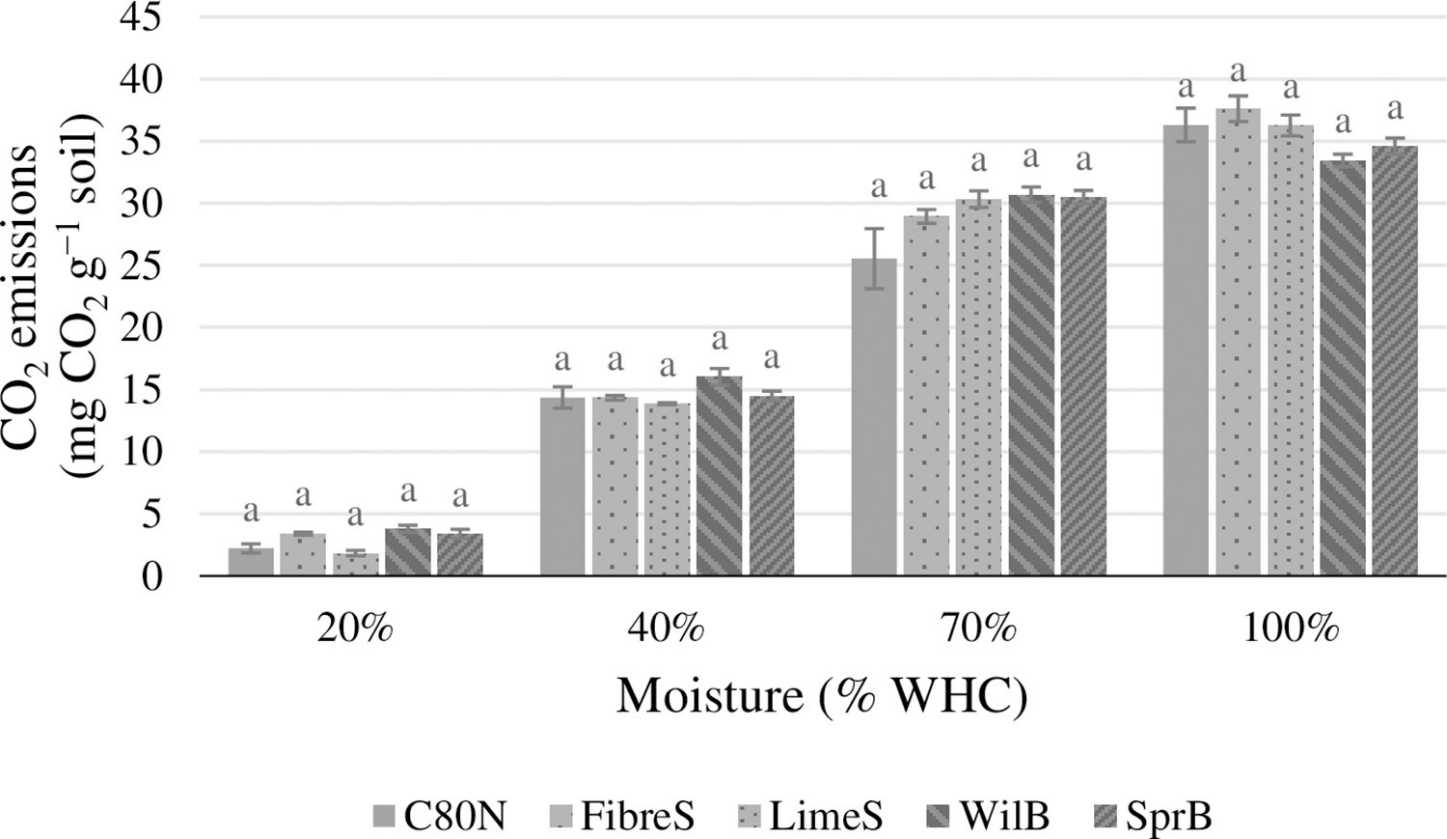
Estimations of the soil CO_2_ emissions (mg CO_2_ g^-1^ soil) over the whole incubation period (33 d) at the four soil moisture levels (20%, 40%, 70%, and 100% WHC). The experimental treatments were: Fibre sludge (FibreS), lime-stabilized pulp sludge (LimeS), willow biochar (WilB), spruce biochar (SprB), and unamended control (C80N). The error bars represent standard errors of the mean (N = 4). Statistical differences (*p*<0.05) are indicated by lowercase letters.

**Fig 3:**
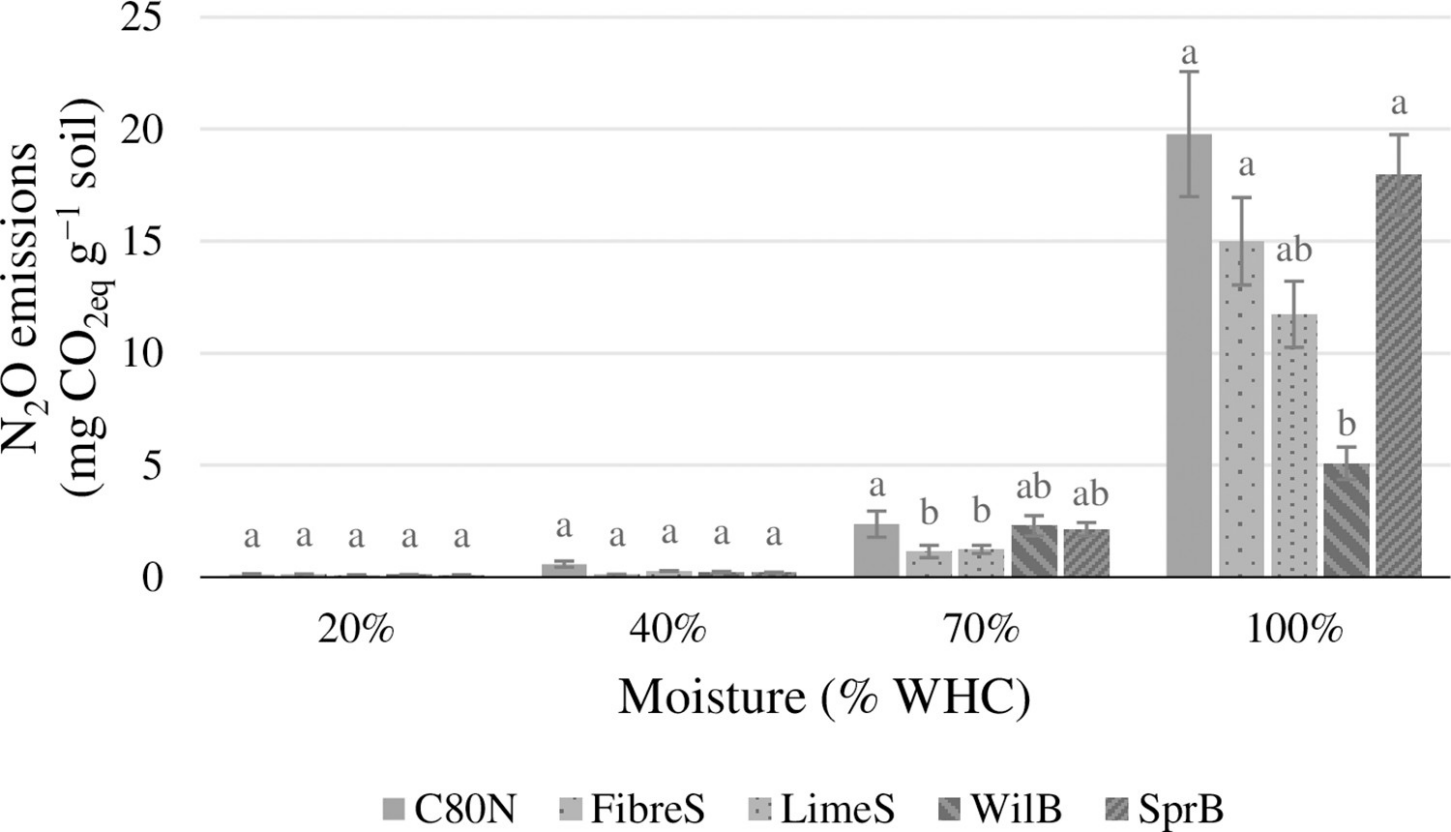
Estimations of the soil N_2_O emissions over the whole incubation period (33 d) at the four soil moisture levels (20%, 40%, 70%, and 100% WHC), and expressed as CO_2_-equivalents (mg CO_2-eq_ g^-1^ soil). The experimental treatments were: Fibre sludge (FibreS), lime-stabilized pulp sludge (LimeS), willow biochar (WilB), spruce biochar (SprB), and unamended control (C80N). The error bars represent standard errors of the mean (N = 4). Statistical differences (*p*<0.05) are indicated by lowercase letters.

**Fig 4:**
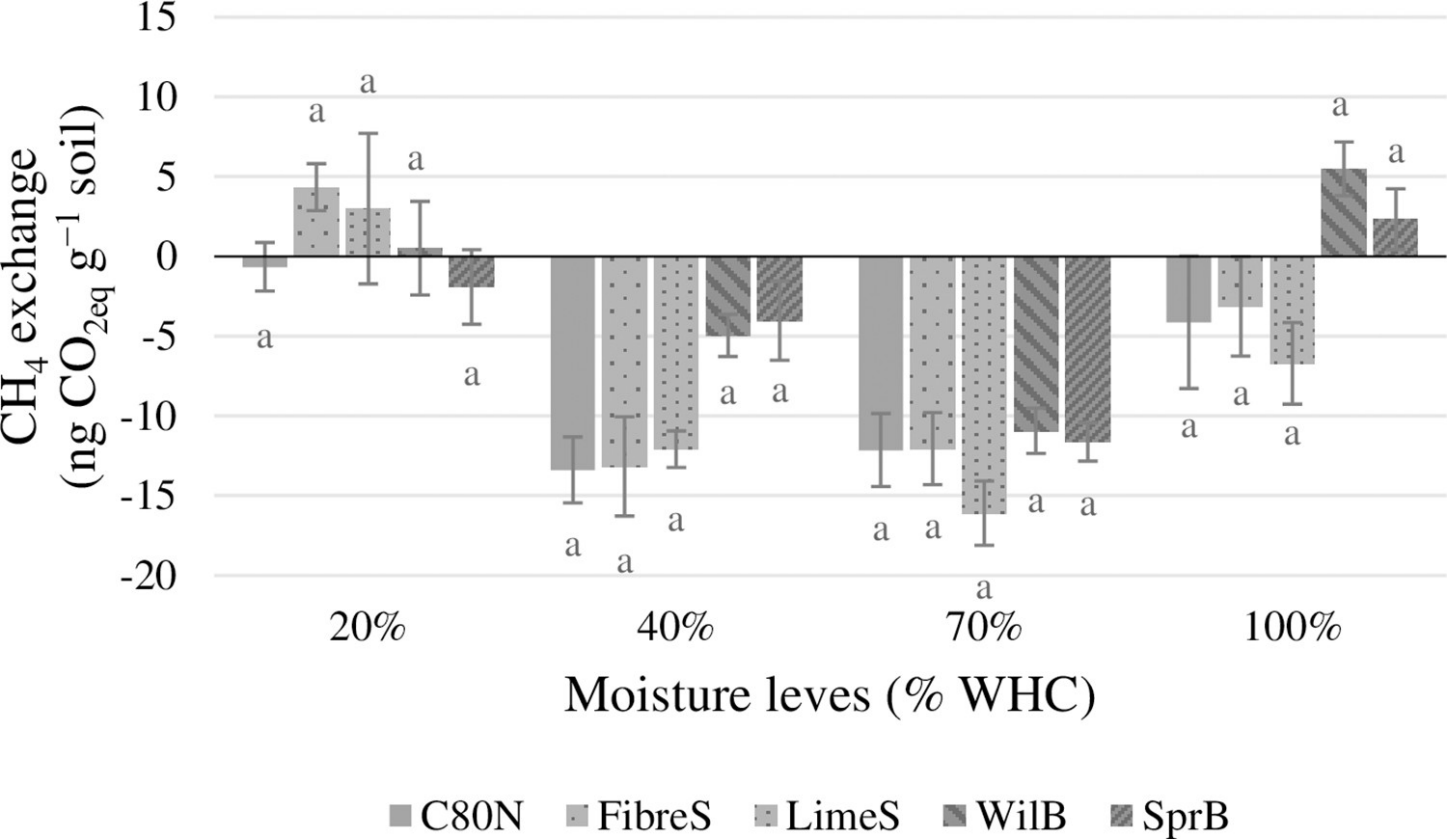
Estimations of the soil CH_4_ exchange over the whole incubation period (33 d) at the four soil moisture levels (20%, 40%, 70%, and 100% WHC), and expressed as CO_2_-equivalents (ng CO_2-eq_ g^-1^ soil). *The experimental treatments were*: F*ibre sludge (FibreS)*, *lime-stabilized pulp sludge (LimeS)*, *willow biochar (WilB)*, *spruce biochar (SprB)*, *and unamended control (C80N)*. *The error bars represent standard errors of the mean (N = 4)*. *Statistical differences (*p*<0*.*05) are indicated by lowercase letters*.

The soil CO_2_ emissions were mostly unaffected by the soil amendments (Fig 2), and there were no significant treatment effects within individual moisture treatments or across all the moisture treatments. In the case of soil N_2_O emissions, we found that FibreS and LimeS produced significantly less N_2_O (*p*<0.05) compared to C80N at 40% WHC, whereas at 100% WHC, only WilB produced less N_2_O (*p*<0.05) than C80N (Fig 3). On average, ligneous soil amendments reduced N_2_O emissions by 13%, 64%, 28%, and 37%, at soil moisture levels of 20%, 40%, 70%, and 100% WHC, respectively. Furthermore, no clear relationships between GHG emissions and soil pore classes were found. However, the N_2_O emissions measured at 20% WHC did exhibit strong negative correlation (R^2^ = 0.92, *p* = 0.03) with large macropores (Ø 0.3 mm; pF 0.0), whereas at 100% WHC, N_2_O emissions had a negative correlation (R^2^ = 0.91, *p*<0.03) with ultramicropores (Ø 1.20 μm; pF 3.4). The CH_4_ exchange did not exhibit any significant treatment effects, and its global warming potential was an order of magnitude smaller than either CO_2_ or N_2_O emissions (Fig 4). Furthermore, all experimental soils were found to be small sinks of CH_4_ under intermediate moisture conditions (40% and 70% WHC) (Fig 4).

**Fig 5:**
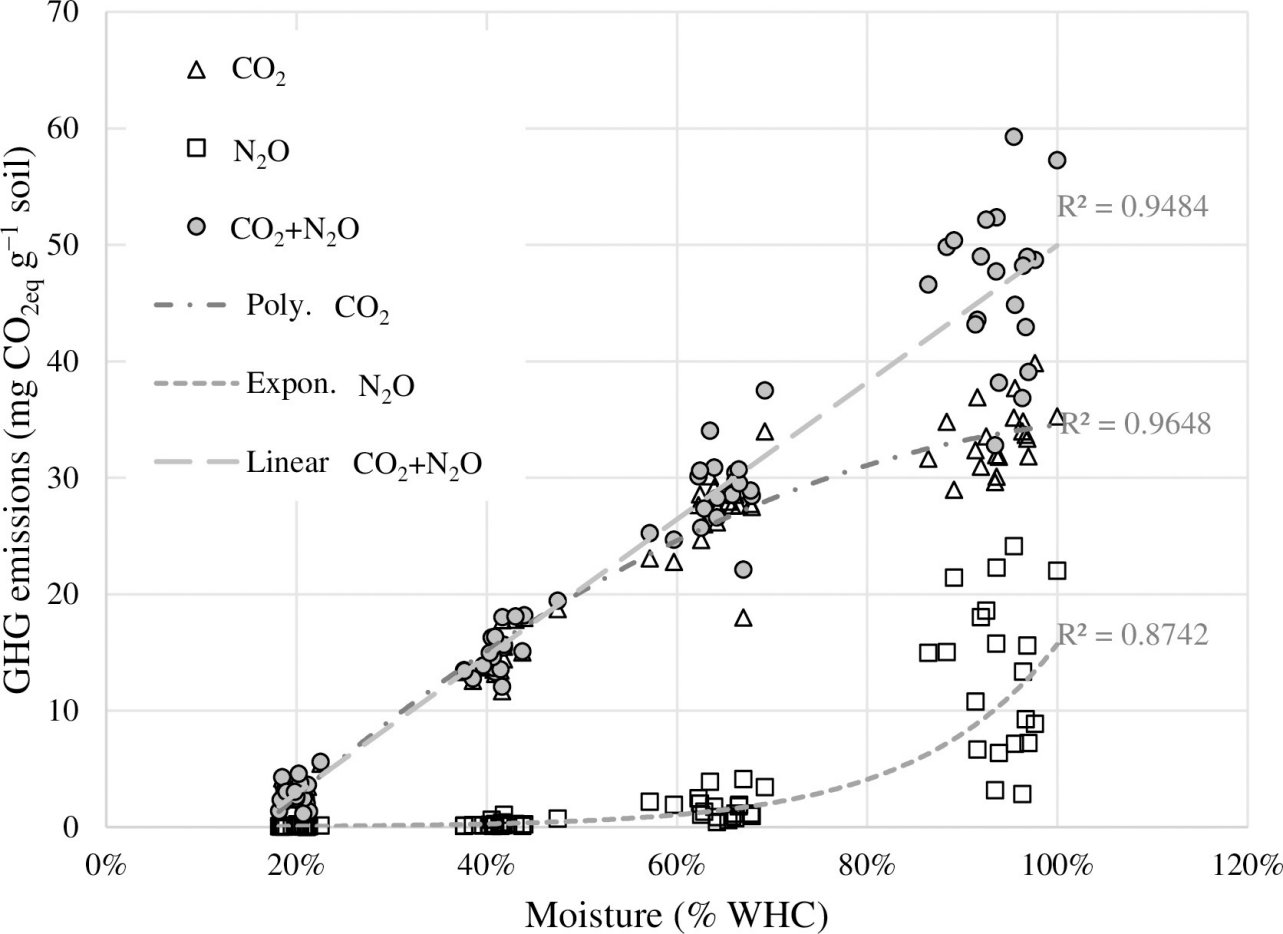
Relationship between CO_2_, N_2_O, and the sum of the two emissions over the whole incubation period (33 d) and soil moisture content. The regression coefficients represent the best fit across all treatments.

The soil CO_2_ emissions (33d) exhibited a strong linear relationship with soil moisture when comparing the CO_2_ production of the moisture treatments at 20%, 40% and up to 70% WHC; thereafter, the rate of increase was slightly reduced (Fig 5). Therefore, the overall relationship was best represented by a polynomial function (R^2^ = 0.96). The N_2_O emissions exhibited a strong exponential (R^2^ = 0.87) relationship with moisture across all treatments (Fig 5). Therefore, when CO_2_ and N_2_O emissions were summed, the combined GHG emissions exhibited a strong linear relationship (R^2^ = 0.95) with moisture (Fig 5).

## 4 Discussion

### 4.1 Soil organic carbon

As hypothesized, all ligneous soil amendments increased SOC content (Table 2), while the magnitude of the increase was comparable to the proportion of recalcitrant C fractions, i.e., lignin-derived C. This assumption is supported by a study by Heikkinen et al. [24], where they determined that pulp sludge biomasses–analogous to the ones in our study–contained 20% to 50% of non-soluble C. This corresponds well with the recovery rates we determined for the pulp sludge treatments in our experiment (Table 2). On the contrary, the recovery rates of biochar in our study were much lower than previously reported [24,75]. Based on previous literature, it is likely that some biochar had been decomposed [76], while a proportion of the remaining recalcitrant C was translocated to deeper soil layers due to tillage and biological processes such as burial by plant roots and earthworms [77,78], although we have no direct evidence of this. Alternatively, the recovery rates of any C inputs may vary significantly depending on sampling practices [79,80], especially in cultivated soils where tilling practices influence soil BD [81]. Nevertheless, recovery rates from 46% to 61% (Table 2) indicate that both lignin-rich pulp sludge and biochar have a significant C sequestration potential [10,69]. The amount of microbial biomass is considered to reflect the total organic matter content of the soil [3], but our study shows that the quality of organic C cannot be overlooked as SprB increased SOC content the most but had slightly less impact on microbial biomass than other amendments. Therefore, future studies should aim to determine whether C sequestration by biochar is more efficient at improving biological indicators of soil health compared to less recalcitrant soil amendments like pulp sludge [82,83].

### 4.2 Soil bulk density and porosity

All treatments exhibited lower soil BD at the amended top soil compared to the unamended subsoil (Fig 1). However, the differences between the two soil layers were not significant when compared to the unamended control. O’Toole et al. [84] estimated that incorporation of 25 Mg C ha^–1^ Miscanthus biochar significantly increased soil porosity, and that mass dilution accounted for only 7% of the reduction in BD of a silty clay loam. We estimate that essentially all of the treatment effects on BD can be explained by the mixing of lighter ligneous biomass with heavier soil particles, indicated by the relationship between the ΔSOC and the ΔBD (S4 Appendix in S1 File). Furthermore, the trend whereby some of the amendments had proportionally less structural pores and more textural pores can be accounted by mixing. As the amendment particles consolidate themselves into the soil matrix, they may increase, reduce, or have no effect on the volume of macropores depending on the size and shape of the biochar particles. However, because of their porous nature they will increase the proportion of smaller textural pores (Table 3 and S3 Appendix in S1 File). Therefore, contrary to our initial hypothesis, the ligneous soil amendments did not improve the overall soil structure by increasing total porosity; they did have a marginal influence on soil pore size distribution, which may be beneficial through improved water retention.

### 4.3 Soil pH

Soil pH is considered an important master variable known to influence soil microbiology [85], and GHG emissions produced by microbial activity [86–88]. In accordance with our hypothesis, the alkaline soil amendments increased soil pH by 0.4–0.8 units compared to the control. Other studies in boreal and temperate regions have reported a similar liming effect with alkaline soil amendments [75,89,90] indicating that ligneous soil amendments can increase and sustain more alkaline soil pH, even though anions, such as bicarbonate, are prone to leaching. We attributed the liming effect to calcium carbonates, and to various hydroxides, oxides or silicates added to the pulp during paper production, or when the pulp sludge is sanitized [91], and to carbonates or alkaline salts (mainly oxides CaO, MgO, K_2_O) enriched during the pyrolysis processes used to produce biochar [92,93]. The relevance of alkaline salts is accentuated by the strong positive correlation between cations and the liming effect, which may revert soil acidification by saturating soil exchange sites with basic cations [25]. Furthermore, the significant liming effect was shared by all treatments, and was not affected by the differences in specific surface area (BET, Table 1), which makes it unlikely that the liming effect was caused by a build-up of carboxylic groups due to surface oxidation, as has been suggested for biochar [94–96]. Therefore, we suggest that the mechanism responsible for the liming effect was related to alkaline salts present in the ligneous soil amendments [25].

### 4.4 Soil greenhouse gas emissions and soil moisture

The soil CO_2_ emissions were not directly affected by the ligneous amendments (Fig 2). This is in line with previous studies involving biochar, which have often reported a lack of significant effects on soil CO_2_ emissions [36,55,97–99]. Furthermore, the magnitude of CO_2_ emissions reflected the fluxes measured as part of a parallel field experiment by Kulmala et al. [100]. However, the determining factor controlling CO_2_ production in our study was soil moisture (Fig 5), which together with temperature and substrate availability determine soil activity in most ecosystems [101,102]. This makes us conclude that the observed changes in soil physical properties did not have a significant effect on soil water regime as we found no differences between soil amendments or the control, except at 70% and 100% WHC, when the CO_2_ emissions from WilB were significantly lower than with the other treatments. This was likely result of an artefact caused by the local soil compaction of the WilB plots, indicated by their significantly higher BD (Table 3). The compacted soil likely facilitated anaerobic conditions, which in turn either inhibited CO_2_ production or limited its diffusion. Less extreme reduction of CO_2_ emissions by high water content at 100% WHC was evident with all treatments, as the CO_2_ emissions increased almost linearly from 20%, 40%, and up to 70% WHC. Thereafter, the CO_2_ emissions continued to increase, but at a reduced rate (Fig 2). On the other hand, N_2_O emissions increased exponentially when comparing the moisture treatments (Fig 3). This dynamic caused the total GHG emissions (in CO_2_-equivalents) to increase linearly throughout the moisture treatments from 20% to 100% WHC (Fig 5). Together the CO_2_ and N_2_O emissions demonstrate a dynamic and rapid shift from aerobic to anaerobic soil respiration in response to being rewetted to different moisture contents.

Previous studies have reported that the optimum soil moisture for N_2_O emissions is in the range of 70–80% of the water-filled pore space [14,103], corresponding to approximately 50–60% of WHC in our study. At higher water contents, diffusion of N_2_O becomes restricted, promoting its reduction to N_2_ [104]. In our incubation experiment, the shallow soil layer, produced by the 50 g of soil may have allowed more efficient diffusion of gases than in undisturbed soil, which could explain why N_2_O emissions continued to increase even above the optimum soil water content. However, efficient diffusion should provide us with a good estimate of N_2_O emissions originating from micropores associated with the soil amendments. Therefore, although our study may overestimate the moisture response of N_2_O production, it should still provide a reliable estimate of the treatment effects.

The importance of pH for soil processes is emphasized by its high correlation with microbial biomass C and N measured before the incubation. However, when measured at the end of the incubation, MBN had a strong correlation with the CO_2_ fluxes along with soil moisture whereas their correlation with MBC was weak. The discordance between MBC and CO_2_ fluxes may be due to methodological factors related to extracting samples with varying water content. However, in our study MBC values were significantly elevated at both moisture extremes (S5 Appendix in S1 File), whereas previous studies have generally reported lower MBC values in a similar context [105,106]. Alternatively, the increased MBC values may indicate microbial stress responses to extreme moisture conditions [107,108]. In our study, MBN and CO_2_ emissions increased in parallel to soil moisture (S6 Appendix in S1 File), whereas N_2_O emissions increased exponentially with soil moisture. We assumed that denitrification was the primary source of N_2_O, as a significant amount of N_2_O was produced only at the highest water content [109,110]. Since denitrification is mainly driven by temperature, NO_3_^–^ concentration as well as C and O_2_ availability [53,111,112], we expected to see higher N_2_O emissions with higher NO_3_^–^ concentrations. However, no increase in N_2_O emissions was observed, even though SprB had almost six times higher NO_3_^–^ concentration than the other treatments [34]. We hypothesize that this lack of clear relationship between soil NO_3_^–^ concentrations and N_2_O emissions may be due to entrapment or physical immobilization of NO_3_^–^ inside the biochar pores [98,113,114], or due to other unknown factors that may have limited the availability of the surplus NO_3_^–^ to denitrifying microbes. Further studies with specific focus on microbiology and sorption mechanisms of biochar are needed to determine the retention mechanism and the availability of NO_3_^–^ to microbial processes. As NO_3_^–^ availability did not seem to control N_2_O emissions, we hypothesize that the reduction in N_2_O emissions observed primarily with the pulp sludge treatments at 70% WHC and at 100% WHC for WilB was caused by a shift in denitrification stoichiometry, i.e., N_2_O/(N_2_O+N_2_) ratio [87,115], which would explain why N_2_O emissions were reduced while MBN and CO_2_ emissions remained unaffected. Even though the specific mechanisms remain speculative [110], several studies have suggested that increasing soil pH may reduce N_2_O emissions [88,116–119] by favouring the synthesis of N_2_O reductase that promotes the complete denitrification, i.e., further reduction of N_2_O to N_2_ [31,116,117,120].

Soil CH_4_ emissions originate predominantly from waterlogged soils or anoxic microsites [121], which are rare in cultivated mineral soils, which often have earation and are artificially drained to maintain optimal moisture conditions. In our study, even a considerably long period (33 days) of water-saturation did not significantly increase CH_4_ emissions (Fig 4).

We acknowledge that the amendments may have led to elevated CO_2_ emissions right after their application, however, based on a parallel field experiment [100] and the present incubation experiment, the studied ligneous soil amendments increased soil C stocks and did not exhibit elevated CO_2_ production two years after their application. This suggests that the studied soil amendments could be used for C sequestration. However, the potential for ligneous soil amendments to reduce soil GHG emissions was shown to be highly dependent on soil moisture conditions, especially in the case of N_2_O emissions, which were reduced mainly in response to being wetted to a high water content. However, the highest N_2_O emissions are often observed from soils at high soil water contents (>80% water-filled pore space) [122], typically after heavy rain events. Therefore, it is possible that ligneous soil amendments may help to reduce N_2_O emissions, especially during peak emissions, for example directly after heavy rain [34,123]. It is also possible that experiment was unable to detect the flush of N_2_O emissions [101], especially in the beginning of the incubation. Consequently, if the ligneous soil amendments had significant effect on the recovery time of soil microorganisms, which is often observed after drying and rewetting [124,125], then it is possible that the total emissions were not reduced, but were lost during the initial peak of GHGs at the beginning of the experiment. While, this would mean that the overall effects on GHG exchange were negligible, and were mainly limited by the amount of available substrates, it could indicate a significant benefit to soil health by increasing resilience against droughts.

Given the differences in soil amendments and their application rates, we conclude that the mechanisms behind these effects are unspecific to any particular amendment alone, but are common through their ability to increase SOC or to alter soil pH. Therefore, other ligneous soil amendments with similar chemical properties as biochar could be expected to have similar effects on soil properties and GHG emissions. We propose that future studies quantifying the long-term effects of soil amendments should at minimum determine their effects on SOC content and soil pH [83]. Furthermore, the alkaline pH and high cation concentration of ligneous soil amendments could indicate a potential for reducing soil-borne N_2_O through liming effects.

## 5 Conclusions

Our goal was to assess the residual effects of four ligneous soil amendments on boreal clay soil. We found that three of the four ligneous soil amendments had enduring effects on soil organic C content and soil pH two years after a single application to a clay soil at a rate of 9–22 Mg ha^-1^. With no clear residual effects on soil CO_2_ emissions, our results suggest that the ligneous soil amendments are appropriate for soil C sequestration. Furthermore, the increase in soil pH due to the long-term liming effect of the ligneous soil amendments appears to have a prominent role in modulating the N_2_O emissions from these soils. However, more research is needed to understand how the properties of ligneous soil amendments affect soil N_2_O dynamics. Overall, the potential for ligneous soil amendments to increase fertility of boreal clay soils, or to mitigate climate change, was mainly related to their ability to sequester C and to raise soil pH while other effects on soil structure and GHG emissions were limited.

## Supporting information

S1 File (DOCX)

S2 File (XLSX)
