# Supplementary material for: Ligneous amendments increase soil organic carbon content in fine-textured boreal soils and modulate N2O emissions
Source: PLoS One. 2023 Aug 10;18(8):e0284092. doi: 10.1371/journal.pone.0284092 (PMC10414678; doi:10.1371/journal.pone.0284092)
Supplement: S1 File — (DOCX) [file pone.0284092.s001.docx]

Supplementary 1. The gravimetric moisture content of the incubated soil samples at the beginning of the incubation and at the time of the five gas sampling (day 1, 5, 12, 20, and 33 of the incubation). Each data point represents the average water content across all treatments presented as percentage of their water holding capacity. The error bars represent standard deviation (N=20).

Supplementary 2. Temperature (average 21.2±0.4°C) and relative air humidity (average 24±7.0%) during each of the five sampling occasions. The error bars represent standard deviation (N=3).

Supplementary 3. Total porosity (φ), and pore size distribution divided into proportion of macropores (>30 µm), micropores (5.0–30 µm), ultramicropores (0.02–5.0 µm) and cryptopores (<0.02 µm). Determined from nine soil cores taken from 0.20–0.25 m soil depth in October 2018. Statistical differences between treatments are shown using lowercase letters. Treatments included an unamended control (C80N) and four ligneous soil amendments: fibre sludge (FibreS), lime-stabilized pulp sludge (LimeS), willow biochar (WilB) and spruce biochar (SprB).

|  | **C80N** | **FibreS** | **LimeS** | **WilB** | **SprB** |
| --- | --- | --- | --- | --- | --- |
| ***φ* (%, v/v)** | 51.0^a^ ±0.76 | 50.1^a^ ±1.17 | 48.9^a^ ±1.42 | 45.7^b^ ±0.63 | 47.9^a^ ±0.70 |
| **Macropores >30 µm (%)** | 18.8^a^ ±3.78 | 14.1^a^ ±4.44 | 12.1^a^ ±2.60 | 16.5^a^ ±3.33 | 15.1^a^ ±3.23 |
| **Micropores 5.0–30 µm (%)** | 4.21^a^ ±1.17 | 5.27^a^ ±1.60 | 4.82^a^ ±2.39 | 5.10^a^ ±1.77 | 4.29^a^ ±0.88 |
| **Ultramicropores 0.02–5.0 µm (%)** | 39.7^a^ ±3.73 | 44.4^a^ ±1.25 | 39.0^a^ ±4.48 | 41.0^a^ ±0.93 | 44.2^a^ ±2.17 |
| **Cryptopores <0.02 µm (%)** | 37.3^a^ ±2.92 | 36.2^a^ ±2.71 | 43.7^a^ ±3.35 | 37.4^a^ ±1.77 | 36.5^a^ ±1.65 |

Supplementary 4. A regression analysis of the observed relationship between the change in soil organic carbon (∆SOC) content and change in soil bulk density (∆BD). The pulp sludge and biochar treatments are divided into separate datasets together with the unamended control treatment.

Supplementary 5. Average microbial biomass carbon (A.) and average microbial biomass nitrogen (B.) at the four moisture levels (20%, 40%, 70%, and 100% WHC) measured after the 33-day long incubation.

Supplementary 6. The CO_2_ emissions across all treatments plotted against incubation moisture on the axis above, and microbial biomass nitrogen (mg N kg^–1^ soil) on the axis below.
